# TransAtlasDB: an integrated database connecting expression data, metadata and variants

**DOI:** 10.1093/database/bay014

**Published:** 2018-02-23

**Authors:** Modupeore O Adetunji, Susan J Lamont, Carl J Schmidt

**Affiliations:** 1 Department of Animal and Food Sciences, University of Delaware, Newark, DE 19716, USA; 2 Department of Animal Science, Iowa State University, Ames, IA 50011-3150, USA

## Abstract

High-throughput transcriptome sequencing (RNAseq) is the universally applied method for target-free transcript identification and gene expression quantification, generating huge amounts of data. The constraint of accessing such data and interpreting results can be a major impediment in postulating suitable hypothesis, thus an innovative storage solution that addresses these limitations, such as hard disk storage requirements, efficiency and reproducibility are paramount. By offering a uniform data storage and retrieval mechanism, various data can be compared and easily investigated. We present a sophisticated system, TransAtlasDB, which incorporates a hybrid architecture of both relational and NoSQL databases for fast and efficient data storage, processing and querying of large datasets from transcript expression analysis with corresponding metadata, as well as gene-associated variants (such as SNPs) and their predicted gene effects. TransAtlasDB provides the data model of accurate storage of the large amount of data derived from RNAseq analysis and also methods of interacting with the database, either via the command-line data management workflows, written in Perl, with useful functionalities that simplifies the complexity of data storage and possibly manipulation of the massive amounts of data generated from RNAseq analysis or through the web interface. The database application is currently modeled to handle analyses data from agricultural species, and will be expanded to include more species groups. Overall TransAtlasDB aims to serve as an accessible repository for the large complex results data files derived from RNAseq gene expression profiling and variant analysis.

**Database URL**: https://modupeore.github.io/TransAtlasDB/

## Introduction 

RNAseq provides a comprehensive view of the transcriptome, and can be used for abundance estimation, and identification of allele-specific expression profiles, alternative splicing, splice junction, novel transcripts and nucleotide polymorphisms (1, 2). The majority of studies adopt RNA sequencing for gene and transcript expression profiling between samples or single cells, by counting the number of mapped reads to a given gene or transcript as an estimation of expression levels (3–6). While RNAseq is primarily applied for gene expression analysis, RNAseq is also a form of exome sequencing and recent studies have used RNA sequencing to detect sequence variation in genes expressed in the sample (7, 8). Several algorithms have been developed to estimate transcript-level expression and the widely accepted methodologies make use of the Tuxedo Suite of programs, which includes *TopHat* and *Cufflink*s (9, 10), or the faster and more memory efficient, *HISAT* and *StringTie* (11). *TopHat* (12, 13) and *HISAT* (14) are reference RNAseq read mapping algorithms, while *Cufflinks* (15) and *StringTie* (16) estimate abundance and differential expression from the alignment files. Another method for differential expression analysis is to count the number of reads overlapping genomic features of interest, using quantification programs like *featureCounts* (17) and *htseq-count* (18) or pseudo-alignments programs like *kallisto* (19) or *Salmon* (20). Read counts are required for a wide range of count-based statistical methods for differential expression or differential binding analyses such as *DESeq2* (21), *edgeR* (22). Although RNAseq is generally applied to gene expression analysis, recent studies have performed comparative analysis of RNAseq with exome sequencing for variant detection analysis and the popularly used variant callers include *SAMtools* (23) and the *Genome Analysis Toolkit* (*GATK*) (24). The different data files generated from RNAseq analyses are typically large and complex, and can be a computational bottleneck, and expensive to store, especially with analyses that involve different sample groups (25).

Current storage programs involve centralizing publicly available datasets from related projects on the web. Such programs either entail a one-line summary of published projects, archives of actual files or an integrative framework of various transcriptome analysis tools or biological databases (26–29). Though these storage options provide a great resource for comparative analysis of related published works, they do not address the limitations most scientists working with ‘big data’ experience, which is storage of the numerous, large analyses result files. Furthermore, assessing such data files in the near future will be a tedious process, leading to either reprocessing the data files or replicating the sample study, wasting time and effort. Thus, there is a need for an organizational framework allowing efficient storage of the different data results and uncomplicated access for retrieval of needed information in a meaningful way to answer biological questions. Given the lack of a uniform standard for data storage and management, resources and techniques for organizing and intelligently interpreting essential information are highly desirable.

We have created a sophisticated system, TransAtlasDB for efficiently storing, organizing and integrating the samples metadata, gene expression profiling and variant analyses results from sequenced samples. This system is a standalone database application that incorporates both classes of database technologies for both archiving and retrieving of various transcriptome analyses results. It serves as an organism-independent sample metadata and transcriptome analysis organizational framework and repository for gene-expression analysis and gene-associated variants, such as single nucleotide polymorphism (SNPs) and insertions and deletions. In addition, the application provides an extensive array of tools for uniform data storage and extraction mechanisms for convenient access to investigate potential patterns or research interests across different RNAseq analysis. TransAtlasDB is designed for (i) archiving sample information; (ii) storing gene expression and variant analysis results; (iii) archiving metadata from the different analysis; (iv) validating data entry; (v) generating data tables for reporting; (vi) downloading viewed data tables; (vii) security and integrity of information; (viii) speed and performance in accessing large amounts of data and (ix) uniform and lossless framework minimizing redundancy.

## System architecture

The main objective of this system is to create a platform for storing gene expression profiling and variant information from transcriptome analysis in a unified format, while maintaining data integrity and a consistent environment for data exploration.

**Figure 1.**
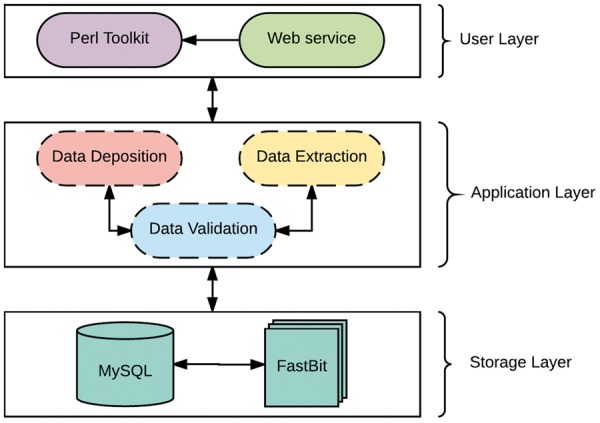
The Architecture of TransAtlasDB.

The system is developed as a client-server architecture, and implemented on a Unix/Linux system. As shown in Figure 1, the system architecture can be divided into three layers: *User, Applicationand Storage* Layer. The *User Layer* offers two modes of interacting with the databases: (i) A PHP interactive web environment with basic access to the databases through the hypertext transfer protocol, and (ii) A command-line Perl suite for interacting with the database. The interactive suite contains pre-configured queries of interest and allowances for custom queries as a print-out or export in user-friendly file formats. In addition, savvy users can interact directly with either database. The *Application Layer* is composed of a suite of Perl scripts and provides an abstraction with a set of procedures for the underlying complexities of parsing, validating, storing and extracting data. This layer is composed of three major components: data validation, data deposition and data extraction. The data validation component ensures all data files slated for storage or extraction are present, the data deposition component executes valid syntaxes for data storage, while the data extraction component functions as a post-processing service for data retrieval and fulfills requests from the User Layer.

The *Storage Layer* is responsible for storing and organizing data using design principles for databases with complex data. Similar to many biological web repositories, we applied a traditional relational data store and due to its availability, simplicity and flexibility, we chose the open source, SQL compliant relational database (RDB) management system, My Structured Query Language (MySQL) (30). This layer has been designed to organize data relationally, employing parent-child key relationships and enabling an efficient management of the stored datasets. Storing data in RDBs provide the convenience of maintaining consistency, data integrity and eliminating redundancy. However, data query performance of RDBs decreases with increased data storage. Given the large amounts of data that transcriptome studies can generate, it is inevitable that query performance will degrade unless alternative solutions are identified. A potential solution involves partitioning the database across a set of machines, which requires often-expensive hardware and will ultimately be cumbersome and expensive to maintain, and, most importantly, not expedient to improving querying performance (31). The requirements for our platform led us to implement a different type of database technology referred to as NoSQL. NoSQL (Not Only Structured Query Language) describes a class of technologies that provides an alternative approach to data storage compared to relational systems; most importantly they do not use relations (tables) as its storage structure and have a schema-less approach to handling large data. Due to the schema-less approach for data storage, NoSQL databases compromises on consistency within the database, and data duplication is allowed which threatens data integrity. Thus, some level of expertise and external protocols are required to adopt some form of data integrity. Regardless, NoSQL database solutions have shown significant advantages on indexing and querying performance with massive amounts of rapidly growing data compared to traditional RDB (32, 33). To ensure availability, simplicity and accessibility for TransAtlasDB, we employed the open source FastBit NoSQL database technology. The FastBit NoSQL database implements a compressed bitmap indexing algorithm for efficient querying of large read-only datasets. It is an append-only and column oriented data store (34). In addition, FastBit has a structured query language (SQL) interface, which guarantees synonymous access to stored data across both database systems. The FastBit SQL algebra provides a unique advantage for simultaneous data archival and retrieval of both the SQL-relational system and FastBit NoSQL system without having to learn another querying language and in turn, the SQL-RDB will enforce data consistency. Consequently, the storage layer is a hybrid model that makes use of both MySQL and the FastBit NoSQL database technology.

TransAtlasDB hybrid architecture is the only independent database system that adopts a novel approach to database storage by the successful integration of both database technology types; RDB and NoSQL. This integration addresses the limitations of using either database systems for the organization and storage of big data, such as the decline in query performance with increasing data stored in RDBs stored is resolved using NoSQL fast access algorithm, and the lack of data integrity and structure with using NoSQL databases is resolved using RDB management systems. Thus, the database is designed to store smaller datasets such as sample information of transcriptome libraries and metadata details from transcriptome analysis in the RDB, while larger datasets such as the variants detected will be stored in the NoSQL system and both records can be queried interchangeably with the benefits of maintaining data integrity and not compromising on querying performance for massive datasets.

### System requirements

The TransAtlasDB database systems were developed using the MySQL Server (v5.5.53) and FastBit (v2.0.3), and designed to work on Unix/Linux operating systems. The software toolkit was written in Perl programming language (v5.18), with required modules listed and freely available on CPAN. The alignment/BAM file mapping parameters were reviewed using the SAMtools package (v1.3.1) (35).

The toolkit and database application were extensively tested by independent parties on the Linux Ubuntu Server (v14.04.5), Ubuntu Desktop (v16.04.2) and Mac OSX (v10.11) operating systems with the latest available MySQL server (v5.7.18), FastBit software (v2.0.3), SAMtools package (v1.5.1) and Perl programming language (v5.22.1). The web interface was written in PHP (v7.1.4), developed using Apache (v2.4.18) and is compatible with most web browsers.

TransAtlasDB source code is available on GitHub at https://modupeore.github.io/TransAtlasDB, and detailed instructions on installation and execution are distributed with the source code.

## Data types

### Input data

TransAtlasDB accepts input data from the different software required for differential expression and variant detection analysis. The current version accepts outputs from the Tuxedo Suite––*TopHat2* or *HISAT2*, *Cufflinks* or *StringTie*, *kallisto*, *Salmon*, *htseq-counts* or *featureCounts*, *SAMtools/BCFtools* or *GATK*. Thus, the following information is required for successful utilization of TransAtlasDB; (a) Sample Information, (b) Alignment information, (c) Expression information and, optionally, (d) Variant information.

The *Sample information*, or metadata, is the reference point of the corresponding results from RNAseq data and therefore important for data archival and retrieval of the various transcriptome analyses. TransAtlasDB preferably accepts the sample information using the FAANG (www.faang.org) sample submission spreadsheet template (https://www.ebi.ac.uk/seqdb/confluence/display/FAANG/Submission+of+samples+to+BioSamples) to BioSamples (https://www.ebi.ac.uk/biosamples) as EMBI-EBI BioSamples has the best support for sample archive. The FAANG sample submission spreadsheet template provides a detailed questionnaire for each sample and hence our database system was modeled to accept the FAANG excel template. However, the required fields in the spreadsheet are the *Animal* and *Specimen* sheets; with the *Animal*-‘Sample Name,’ *Animal*-‘Organism,’ *Specimen*-‘Sample Name’ and *Specimen*-‘Organism Part’ column filled. The database system also accepts a tab-delimited file with the minimum required columns of ‘Sample Name,’ ‘Derived from,’ ‘Organism’ and ‘Organism Part,’ additional columns ‘Sample description,’ ‘First name,’ ‘Middle Initial,’ ‘Last name’ and ‘Organization’ are also accepted. Definition of accepted columns is given in Table 1. Otherwise, the sample information can be manually inserted using SQL insert statements.

**Table 1.** Column names requirement status for Sample information tab-delimited file

Header	Status	Description
Sample name	Required	Sample identification number
Sample description	Optional	Sample description
Derived from	Required	Animal identification number
Organism	Required	Organism name
Organism part	Required	Tissue name
First name	Optional	Person’s first name
Middle initial	Optional	Person’s middle Initial
Last name	Optional	Person’s last name
Organization	Optional	Organization

The *Alignment information* is comprised of the alignment BAM file, summary statistics file and optionally the bed files obtained from RNAseq read mappers, TopHat2 or HISAT2. The *Expression information* consists of the genes normalized abundance files generated using Cufflinks, Stringtie, Kallisto or Salmon containing either or both normalization procedures; Fragments Per Kilobase of transcript per million (FPKM) and Transcripts per million (TPM), and actual feature read counts using HtSeq-count, featureCounts or STAR quantMode option. The *Variant information* includes the variant variant call format (VCF) file (36) from variant callers, such as GATK (24), SAMtools(23) and many more. Table 2 provides an overview of applicable programs accepted in TransAtlasDB.

**Table 2:** List of programs accepted in TransAtlasDB.

Information	Programs
Alignment Information	
	TopHat2
	HiSAT2
	STAR
Expression Information	
	Cufflinks
	Strintie
	Kallisto
	Salmon
ReadCount information	
	htseqcount
	featureCounts
	STAR quantMode
Variant Information	
	GATK
	SamTools
Variant Annotation Information	
	VEP
	Annovar
Sequence files details (optional)	
	FastQC

**Figure 2.**
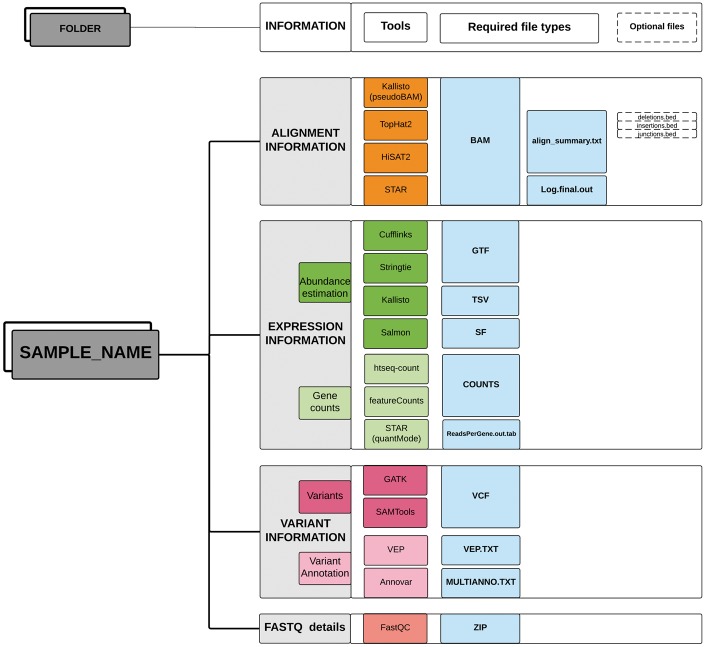
Directory structure layout for each sample. Output files (sufflix) required from the specified software for the different RNAseq analyses data types.

Optionally, the functional annotations of variants predicted by different bioinformatics tools can also be provided in a tab-delimited format. TransAtlasDB currently accepts variant effect annotations from two annotation software; Ensembl Variant Effect Predictor, commonly known as *VEP* (37) and *ANNOVAR* (38). The input data should be stored in a single folder for each sample (Figure 2).

### Output format

TransAtlasDB outputs user-defined queries as a tab-delimited table. This table is the default output format which is accepted by most text editors or statistics tools such as Microsoft Excel, R and JMP software. Aside from the tab-delimited format for exporting results, the variant information can be generated as a VCF output. Predicted functional annotations and sample metadata are added in the INFO field of the VCF file, using the key ‘CSQ’ and ‘MTD,’ respectively. Data fields are separated by ‘|’; the order of fields is written in the VCF header. VCFs produced by TransAtlasDB follow the standard VCF version 4 file format, and can be used for further downstream analysis or visualization using various variant viewers such as the University of California Santa Cruz Genome Browser (39), Integrative Genomics Viewer (40) and other programs that accept VCF files.

## Database structure

The database system is structured using the RDB for the metadata information, alignment information, expression information and summary of the variant information and NoSQL for the variant information. To maintain a coherent archive, the relational approach has been applied to design the basic database concept. The sample information is mapped into the relational table and the sample name will serve as the unique identification number (Id). The Id is used as the primary key for rapid indexing and enforcement of uniqueness, and the table data can be parsed using binary searching procedures across the different data types. The database design ensures mutual table relationships, and centralized checking of the foreign key constraints enforces the referential data consistency and integrity across tables. The parent-child relationships are specified by matching the primary key of the parent table to the child tables.

### RDB (MySQL) schema

**Figure 3.**
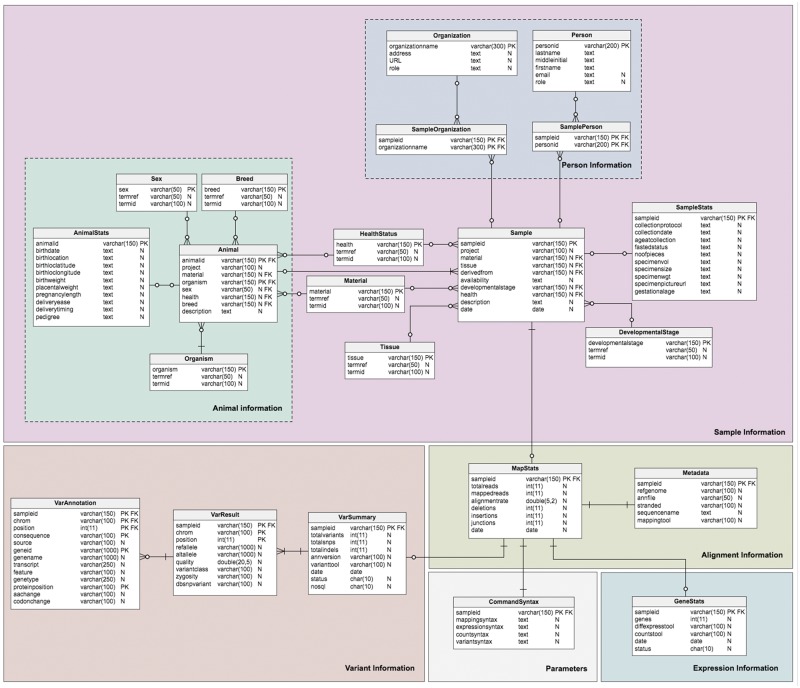
Schema of the TransAtlasDB RDB system. The MySQL tables are grouped by data stored (i.e. Sample Information, Alignment Information, Expression Information and Variants Information).

The database model (Figure 3) is divided into four sections corresponding to the different type of data required: sample information, alignment information, expression information and variant information. This forms a logical and simple organization of the data. The schema contains twenty-one (21) tables, six (6) views and four (4) stored procedures, which are relevant to the organization of the different required datasets; the sample (*Sample* table) and additional information about the sample are stored in the Sample sub-tables. Transcriptome analysis results stored in the alignment summary and statistics (*MapStats* table), and the mapping metadata (*Metadata* table) are one-row descriptions for each sample and alignment details. The expression information summaries are stored in the *GeneStats* table, while the gene expression levels are stored in the NoSQL database. Variant details are organized in the *VarSummary*, *VarResult* and *VarAnnotation* tables. Table 3 provides a brief description of the TransAtlasDB RDB schema.

**Table 3:** Description of MySQL database schema. The MySQL schema consists of (A) 23 tables, (B) 6 views and (C) 4 stored procedures relevant for the organization of the different data sets generation from transcriptome analysis.

Attributes	Description
A. TABLES	
Animal	Animal information
AnimalStats	Additional information on Animal
Breed	Organism Breed
CommandSyntax	Analysis data commands
DevelopmentalStage	Organism developmental stage
GeneStats	Expression information summary
HealthStatus	Organism health status
MapStats	Alignment information and statistics
Material	Type of Sample
Metadata	Alignment information summary
Organism	Organism information
Organization	Organization of scientist
Person	Scientist information
ReadCounts	Raw counts details
Sample	Sample information
SampleOrganization	Cross reference of Sample and Organization
SamplePerson	Cross reference of Sample and Person
SampleStats	Additional information on Sample
Sex	Sex of Organism
Tissue	Organism part
VarAnnotation	Variant annotation information
VarResult	Variant information
VarSummary	Variant information summary
B. VIEWS	
vw_nosql	Sample details
vw_nosql	Prototype of NoSQL template
vw_sampleinfo	Summary analysis and statistics of each sample
vw_seqstats	Sequencing Metadata of all RNAseq analysis
vw_vanno	Variant annotation details
vw_vvcf	Prototype of VCF template
C. PROCEDURES	
usp_vall	Variants information in organism
usp_vchrom	Variants information of a chromosome
usp_vchrposition	Variants information of a chromosomal region
usp_vgene	Variants information of a gene

### Non-RDB (NoSQL) schema

In order to prevent poor query performance in the RDB due to the large volumes of data stored, our current system implements the NoSQL database, FastBit, for archiving of both the gene-expression analysis and gene-associated variant analysis results, using custom transfer protocols from MySQL to the FastBit system. FastBit stores data as tables with rows and columns, and makes an index for each column instead of each row as in RDBs. Thus, the expression and variant information are organized with the same corresponding field names as depicted in the RDB (Table 4). This naming scheme allows a fluid interchangeable interaction with both the MySQL and FastBit platform.

**Table 4.** Fields in FastBit system for querying

Fields		Type	Description
A.			
	sampleid	text	Sample Id
	chrom	key	Reference chromosome
	position	int	Reference Position
	refallele	char	Reference allele
	altallele	char	Alternate allele
	quality	double	Variant Quality
	dbsnpvariant	text	dbSNP membership number
	variantclass	key	Type of variant
	zygosity	key	Genotype
	source	text	Source of annotation
	consequence	text	Variant consequence
	geneid	text	Gene Id (from NCBI or Ensembl)
	genename	text	Gene short name
	transcript	text	Transcript Id (if provided)
	feature	text	Feature annotation
	genetype	text	Location of variant
	proteinposition	int	Relative position of aminoacid in protein
	aachange	text	Aminoacid change
	codonchange	text	Alternative codon with the variant
	organism	text	Organism name
	tissue	text	Tissue
B.			
	sampleid	text	Sample Id
	chrom	key	Gene/Feature chromosome
	start	int	Gene/Feature start position
	stop	int	Gene/Feature end position
	genename	text	Gene short name (if available)
	geneid	text	Gene Id(s) associated with the gene/feature
	coverage	double	Estimated absolute depth of read coverage for the gene/feature
	tpm	double	TPM of the Gene/Feature
	fpkm	double	FPKM of the Gene/Feature
	fpkmconflow	double	The lower bound of the 95% confidence interval on the FPKM of the Gene/Feature
	fpkmconfhigh	double	The upper bound of the 95% confidence interval on the FPKM of the Gene/Feature
	fpkmstatus	char	Quantification status for the Gene/Feature
	genename	text	Gene short name
	tissue	text	Tissue
C.			
	sampleid	text	Sample Id
	genename	text	Gene short name (if available)
	readcount	Int	Read counts per Gene
	organism	text	Organism name
	tissue	text	Tissue

FastBit fields are similar to the (A) variant information tables, (B) expression information tables and (C) gene counts information in the RDB, allowing synonymous access to queries data across both systems.

## Package toolkit

### Package components

TransAtlasDB system provides a command-line toolkit and can be used on diverse hardware systems where standard Perl modules and the Perl-DBD module are installed. The toolkit contains a suite of Perl scripts for handling the varied and large amounts of data generated from gene expression profiling and variant detection analysis. The suite serves several purposes: data entry into and data retrieval from the database(s); data browsing, double entry data validation, and verification of the different data files specified; completeness of data import; generation of complex reports and exports dynamic user-defined queries and extracts subsets of data in tab delimited format. This suite provides semi-automated solutions that simplify the complexity of data storage and data querying methods by creating a user-friendly data management workflow. A brief outline of the package design is provided in Table 5.

**Table 5.** Scripts within the TransAtlasDB toolkit

File Name	module	Description
*INSTALL-tad.pL*		Database installation module
*connect-tad.pL*		MySQL & FastBit re-connection application
*tad-import.pl*		
	metadata	Database sample import module
	data2db	Database import module
	delete	Database sample delete module
*tad-interact.pl*		Database interactive module with pre-configured database queries
*tad-export.pl*		
	query	User database queries
	db2data	Database retrieval module
*example/*		Folder with sample files

Additionally, the package offers a simple and quick installation procedure for setting up the database systems (via INSTALL-tad.pL). The detailed description of the source code and suite functionality with an example of usage are accessible via https://modupeore.github.io/TransAtlasDB/tutorial.html. The basic hardware and software requirements, short instruction of the installation and some test files are distributed within the package directory.

### Toolkit usage

**Figure 4.**
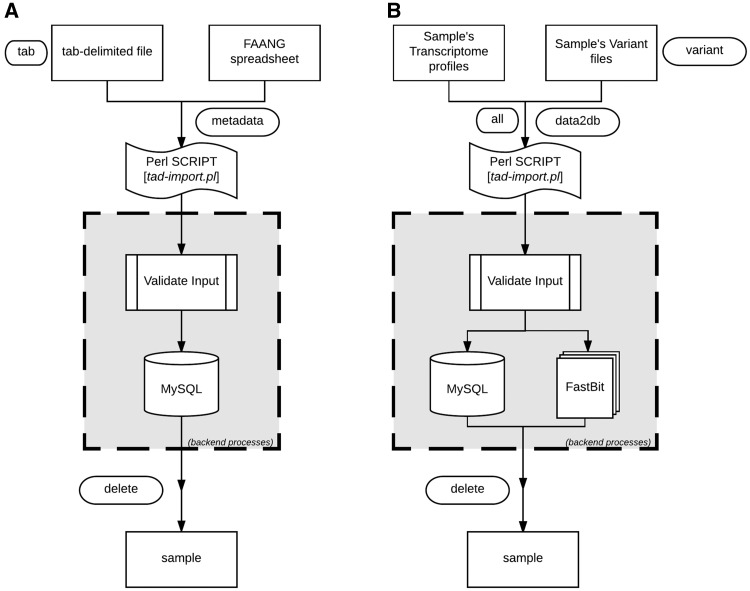
Data import procedure using tad-import.pl and available options for (**A**) samples metadata and (**B**) RNAseq data, respectively.

**Figure 5.**
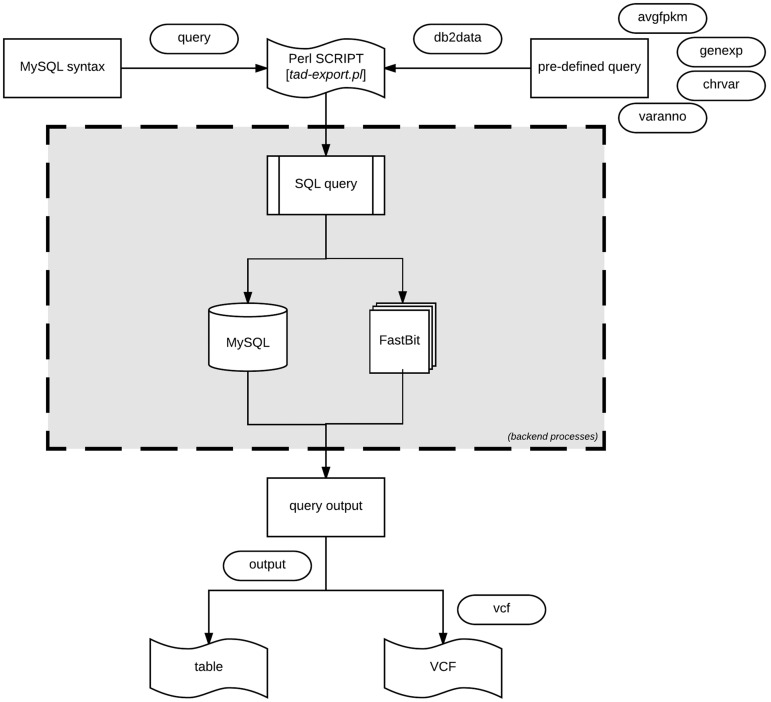
Data export procedure using tad-export.pl and available options either executing a MySQL query syntax or choosing from the four defined (avgfpkm, genexp, chrvar and varanno) options.

TransAtlasDB Perl toolkit (Table 5) is a user-friendly framework that inputs, organizes, validates, archives and process complex transcriptome analyses data. The summaries of every transaction will be stored in log files for future reference. A pictorial representation of the procedures for data import and export are shown in Figures 4 and 5, respectively.

#### Installation of TransAtlasDB

The TransAtlasDB database system and necessary components need to be installed to a local disk using *INSTALL-tad.pL*. The MySQL server and FastBit software should have been previously installed and added to the systems’ or users’ executable path. Only the ‘–password’ argument is required if the user has admin privileges to the MySQL server. Otherwise, additional arguments, such as the ‘–username,’ ‘–databasename’ will be needed. The NoSQL folder-name and location can be optionally specified; if not done, a default folder ‘*transatlasfb*’ will be created in the working directory. The installation module needs to be carried out once per local disk to prevent database access conflict. However, if such conflict arises user settings can be viewed and, if needed, corrected (via *connect-tad.pL*).

#### Import data using tad-import.pl

The sample information or sample metadata consists of the relevant details needed to uniquely identify each specimen used for RNAseq. The sample metadata can be imported (via the ‘–metadata’ argument) from either the FAANG sample submission spreadsheet or a tab-delimited file (Figure 4A). The sample name must be unique for each sample and should follow the sample-naming-scheme of the FAANG BioSamples group––short species code, laboratory or institute short name and alphanumeric sample ID – separated by underscore. For instance, the sample name, *GGA_UD_1004*, represents *Gallus gallus* species from University of Delaware with sample ID 1004. The sample information is also the reference point for the resulting data from transcriptome analysis with such sample.

After importing the sample metadata, the transcriptome analysis results can be inserted using the ‘–data2db’ argument (Figure 4B). The transcriptome profiling data or variant analysis data can be imported together (‘–all’ flag) or separately (‘–gene’ or ‘–variant’ flag) with data files in the directory structure presented in Figure 2. The variant functional-annotations predicted from either VEP or ANNOVAR can also be imported using additional flag (‘–vep,’ ‘–annovar,’ respectively) and must be in their default tab-delimited format. If VEP, the filename should end with ‘.vep.txt,’ or else if ANNOVAR, the file having suffix ‘.multianno.txt’ will be accepted.

Be aware that analysis results for a sample can only be imported once to ensure data integrity, nonetheless, previously imported data can be cautiously deleted using the ‘–delete’ argument followed by sample name.

#### Export data using tad-export.pl

The export module offers two methods of extracting data from the database. One method allows users to execute direct data manipulation language SQL statements to the RDB (using the ‘–query’ argument). For instance, executing the query ‘show tables’ will retrieve all the rows currently in the database, which can be stored as a tab-delimited file.

The second method (via ‘–db2data’ argument) consists of four options that are of research interest: (1) Average expression values of specified genes organized by the different tissues. (2) Gene expression profiles across the different samples of the same organism. Specific samples can be selected. (3) Variant distribution of all, or selected chromosomes for individual samples in the database. (4) Variants and predicted functional annotations found in the organism or selected genes or chromosomes. The exported results can be written as a tab-delimited table or VCF output for variants (Figure 5).

If uncertain how to proceed with the export module, the interaction module (via *tad-interact.pl*) provides an easy-to-use menu-driven interface. The menu offers seven choices of exploratory research interest and provides a detailed description of what can be done from the module. With little effort, it is self-explanatory to use. The interaction module only displays a small subset of results, nevertheless, further instructions on how to export the complete results will be displayed.

### Web portal and use cases

The PHP web environment provides another user-friendly access to the TransAtlasDB database system. The web portal provides detailed overview of the samples currently archived in the system, and options to query and export requested data from the database system. It relies on the perl command-line toolkit for interacting with the databases.

The use cases below are some examples of how biological inferences can be derived from the various RNAseq analysis data files stored in our TransAtlasDB system using the web environment. These examples can also be retrieved in the command-line toolkit provided.

TransAtlasDB web interface is comprised of five sections: (i) About page gives a summary of the samples archived in the database. (ii) Data Import page provides two methods of importing the samples metadata; either by uploading a sample file (FAANG spreadsheet or template tab-delimited file) or by manual entry. Storing the large data files such as the gene expression profiling and variants analysis results can only be done using the Perl toolkit as explained above. (iii) SQL Query page executes specified SQL queries to both databases. (iv) Metadata page displays the samples stored in the database and an overview of each sample storage-status as well as the analysis summary where applicable. The samples can be exported as a tab-delimited file. (v) Gene Expression page; the gene expression data can be viewed based on the individual sample-gene expression or average expression profiles of multiple genes across all samples and tissues. By specifying one or more genes by their gene symbols, a fuzzy search is performed based on the characters specified. (vi) Variant page; variants can be viewed through querying gene symbols or chromosomal regions.

#### SQL queries

**Figure 6.**
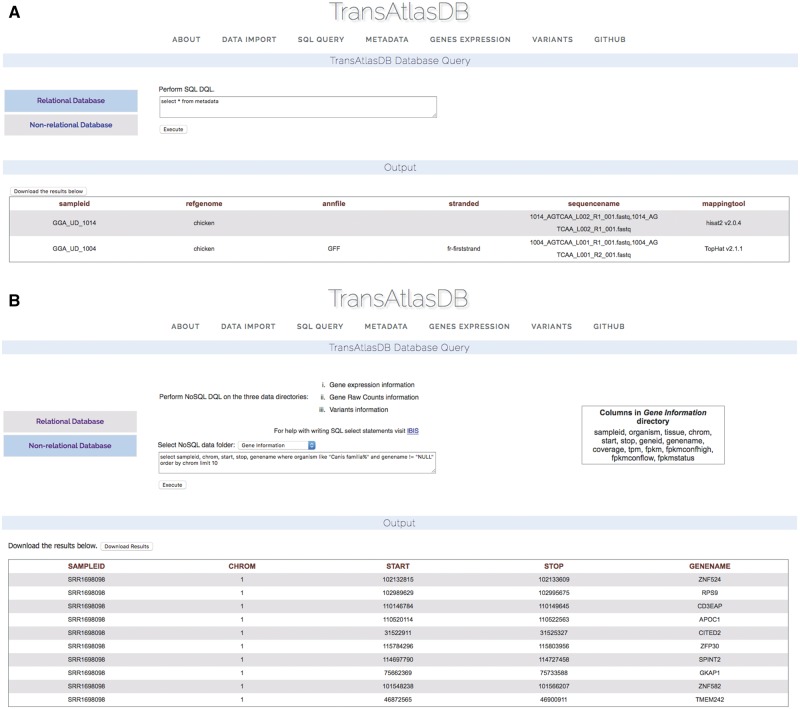
Performing SQL DQL via the web interface to the (**A**) relational and (**B**) non-RDB.

TransAtlasDB allows for the execution of SQL data query language (DQL) to both the MySQL RDB and FastBit NoSQL database using the appropriate SQL DQL syntaxes. This feature provides users unrestricted access to both database content without the limitation of having to interact with the command line. Select statements performed on either database will return a table of records based on the select expression. For instance, viewing the mapping metadata of all samples stored in TransAtlasDB; executing ‘select * from Metadata’ will provide all records stored in the *Metadata* table (Figure 6A). FastBit provides an interactive bitmap index search which are identical to SQL DQL statements but recognizes a limited number of attributes compared to the RDB. Select statements executed to the NoSQL directories will not require the FROM clause to be specified, rather the NoSQL directories can be selected from the drop-down menu provided. Figure 6B displays the *gene-information* records for this statement: ‘select sampleid, chrom, start, stop, genename where organism like ‘Canis familia%’ and genename! = ‘NULL’ order by chrom limit 10.’

#### Summary of TransAtlasDB content and analyses metadata

**Figure 7.**
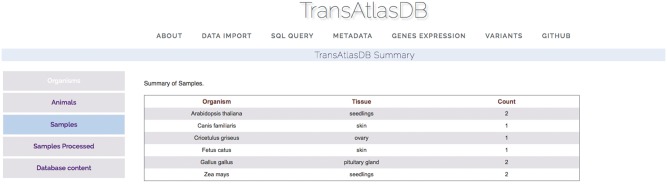
Various summary tables displaying database content in the About page.

**Figure 8.**
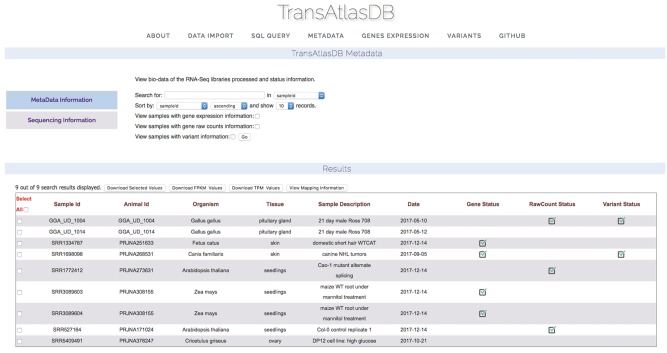
Status of analysis data previously archived.

Descriptive tables of the database content and status of the analyses data import can be displayed to provide users a way to quickly visualize samples already archived in the database and get the current status of all the samples archived in the database. Summary tables can be viewed in the About page (Figure 7), and Analyses data import status tables can be viewed in the Metadata page (Figure 8).

#### Investigating gene expression levels and variants

**Figure 9.**
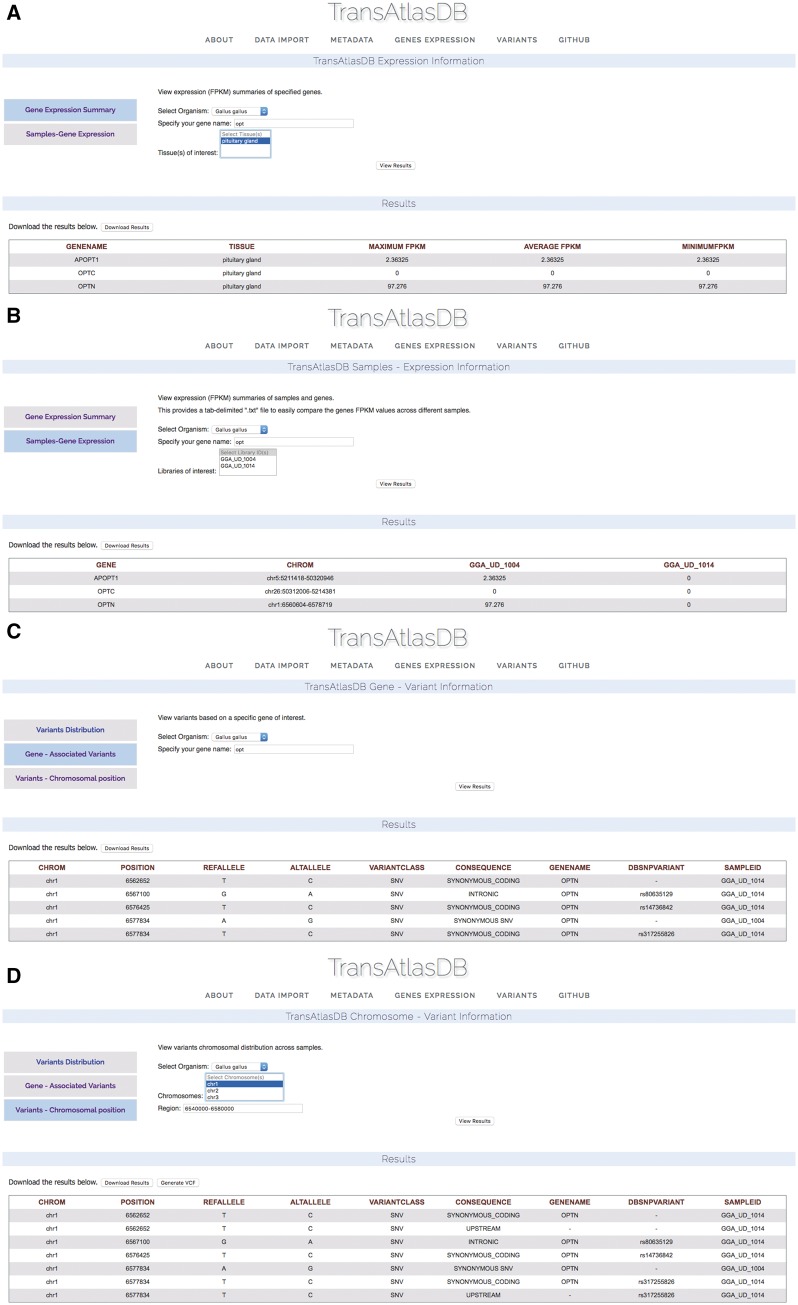
Use Cases via the web interface. (**A**) Genes summary expression levels across all samples. (**B**) Genes fpkm expression level for each sample. (**C**) Variants found in the OPTN gene. (**D**) Variants found around the chromosomal region of the OPTN gene.

Based on the example data files provided, two Gallus *gallus* samples from the Pituitary gland, were previously imported into TransAtlasDB. Consider examining a summary of the Optineurin (OPTN) gene from the samples in the database (Figure 9A) on close inspection the summary of OPTN reveals identical minimum, average and maximum fpkm values indicating the gene may have identical fpkm values across all samples. Further exploration based on individual samples reveals OPTN may not be expressed in one of the samples, GGA_UD_1014, despite being obtained from the same tissue, Pituitary gland (Figure 9B). These results can be exported as a tab-delimited file and adapted into statistical packages such as R or JMP for further analysis.

Unsure of the reason for different expression of the OPTN gene between the two samples, the variants can be examined by specifying the gene name (Figure 9C) or chromosomal region (Figure 9D). Multiple synonymous SNPs were found along the OPTN genomic region in sample GGA_UD_1014, while GGA_UD_1004 had only one synonymous SNP. The large number of SNPs in the GGA_UD_1014 sample, though synonymous, may have an effect on gene expression or mRNA stability and serves as a potential avenue for further analysis. These results can also be exported as a tab-delimited file for statistical analysis or as a VCF file to be used for downstream analysis or visualization.

## Future developments

The limitless resource potential of TransAtlasDB provides numerous options for expansion to integrate data files from other transcriptomic analyses studies and other next generation sequencing platforms like Exome sequencing. The database system is currently being extended to integrate analyses data files from human cancer studies.

## Conclusion

The TransAtlasDB system should serve as a useful management platform for samples metadata and data derived from transcriptome analyses. Users can expertly store sample information and RNAseq analyses results and retrieve needed data based on specified query either using the Perl toolkit or web environment provided. TransAtlasDB provides the abstract layer with methods for data manipulation with minimum efforts to install a running system. TransAtlasDB adopts a hybrid infrastructure containing both types of database technologies; the RDB system maintains data in an organized form that eliminates data redundancy and enables efficient data management while the NoSQL system provides fast indexing and query performance that scales beyond the capabilities of RDBs. This makes TransAtlasDB a sophisticated database system capable of storing, organizing and maintaining massive and complex transcriptome analyses data without compromise in performance whilst enforcing data integrity. The modular architecture of the system makes it possible to expand and integrate other analysis procedures potentially needed in the future. The database application is currently modeled to handle analyses data files from agricultural species, and will be expanded to include more species groups. A major advantage is that the platform can be installed locally, where users can personalize the hardware/software environment and data to import for storage, organization, access and exchange of biological data. The modular architecture of the toolkit also enables addition of any extensions needed by the user. It is believed TransAtlasDB will be a useful and user-friendly environment for transcriptome analyses database storage.

## Funding

This project was supported by Agriculture and Food Research Initiative Competitive Grant 2011-67003-30228 from the United States Department of Agriculture National institute of Food and Agriculture.

*Conflict of interest*. None declared.
